# Retraction: Enhancing stroke disease classification through machine learning models via a novel voting system by feature selection techniques

**DOI:** 10.1371/journal.pone.0324683

**Published:** 2025-05-20

**Authors:** The PLOS One Editors

The *PLOS One* Editors retract this article [1] due to concerns about:

- Compliance with PLOS policies on Manipulation of the Publication Process and Authorship
- Reliance on retracted work cited in References 5, 18, and 21
- The use of terminology that differs from standards in the field (e.g. “genuine positive” instead of “true positive”)

MH, FY, and MMH responded but expressed neither agreement nor disagreement with the editorial decision. XY, SY, HJ, and SMSI either did not respond directly or could not be reached.
